# Progress in the Correlative Atomic Force Microscopy and Optical Microscopy

**DOI:** 10.3390/s17040938

**Published:** 2017-04-24

**Authors:** Lulu Zhou, Mingjun Cai, Ti Tong, Hongda Wang

**Affiliations:** 1 State Key Laboratory of Electroanalytical Chemistry, Changchun Institute of Applied Chemistry, Chinese Academy of Sciences, Changchun 130022, China; llzhou@ciac.ac.cn (L.Z.); caimingjun@ciac.ac.cn (M.C.); 2 University of Chinese Academy of Sciences, Beijing 100049, China; 3 The Second Hospital of Jilin University, Changchun 130041, China

**Keywords:** atomic force microscopy, conventional florescence microscopy, super-resolution fluorescence microscopy, correlation

## Abstract

Atomic force microscopy (AFM) has evolved from the originally morphological imaging technique to a powerful and multifunctional technique for manipulating and detecting the interactions between molecules at nanometer resolution. However, AFM cannot provide the precise information of synchronized molecular groups and has many shortcomings in the aspects of determining the mechanism of the interactions and the elaborate structure due to the limitations of the technology, itself, such as non-specificity and low imaging speed. To overcome the technical limitations, it is necessary to combine AFM with other complementary techniques, such as fluorescence microscopy. The combination of several complementary techniques in one instrument has increasingly become a vital approach to investigate the details of the interactions among molecules and molecular dynamics. In this review, we reported the principles of AFM and optical microscopy, such as confocal microscopy and single-molecule localization microscopy, and focused on the development and use of correlative AFM and optical microscopy.

## 1. Introduction

Atomic force microscopy (AFM) was invented by Binnig et al. in 1986 [1]. AFM is a powerful tool to provide various information via detecting the weak interactions between the tiny tip on a cantilever and the sample surface. Compared to optical microscopy and electron microscopy (EM), AFM overcomes the wavelength limitations of light and electrons, and is capable of acquiring three-dimensional topography, molecular interactions and dynamics under various conditions (vacuum, atmosphere, and liquid) [2]. Since AFM has the abilities of high-resolution imaging and high-sensitivity detecting, it has been extensively applied to various areas, such as life science, surface science, and material science [3,4].

AFM has the unique ability to investigate biological samples without labelling or fixation in their native conditions at the single-molecule level. By modifying the AFM tip with specific ligands, such as antibodies and lectins, we simultaneously acquire the topographic and recognition image of a biological sample, which reveals the localizations of specific receptors on the sample surface; we also acquire various information (stiffness, elasticity, and unbinding force) of the ligand-receptor interactions using the AFM technique [5,6,7]. However, it is difficult to locate two or more types of components on the cell membranes by the AFM technique; and AFM cannot be used to image intracellular molecules because it is only used to investigate the sample surface.

Optical microcopy is an indispensable tool in biological research. By labelling samples with fluorophores, optical microscopy provides a way to identify specific components and to investigate interactions among different components. Due to the diffraction limit of light, conventional fluorescence microscopes, such as confocal laser scanning microscopy (CLSM) and total internal reflection fluorescence microscopy (TIRFM), have a limited resolution—a lateral resolution of about 250 nm and a vertical resolution of about 500 nm—and, thus, conventional fluorescence microscopy cannot be used to investigate biological samples at the single-molecule level [8]. Fortunately, several super-resolution imaging techniques, such as stimulated emission depletion (STED) [9], stochastic optical reconstruction microscopy (STORM) [10], and photoactivated localization microscopy (PALM) [11], have been developed and shattered the diffraction barrier; for example, STED can achieve a lateral resolution of 20–70 nm and a vertical resolution of 40–150 nm; single-molecule localization microscopy, such as STORM and PALM, can achieve a lateral resolution of 10–30 nm and an axial resolution of 10–75 nm [12]. These super-resolution fluorescence microscopy techniques can also provide an excellent opportunity to study the distributions of specific components and the interactions among different components at nanometer resolution. Although optical microscopy has abilities to simultaneously identify several specific components and to deeply visualize a cell, it cannot provide the high-resolution topography of the cell which reveals the cell structure.

To complement the advantages and shortcomings of both techniques, we can combine AFM with optical microscopy to provide more information. The combination of two or more complementary techniques has currently become a hot topic in the research of novel multi-functional instruments. Recently, *Nature* highlighted the correlative microscopies, stating two microscopes are better than one [13]. At present, several correlative microscopy techniques, such as optical microscopy/EM and optical microscopy/AFM, have been developed and achieved gratifying results [14,15]. In this review, we will describe the main principles of AFM and optical microscopy, and summarize the progress of correlative optical microscopy/AFM techniques in biological research.

## 2. Principles of Atomic Force Microscopy and Optical Microscopy

### 2.1. Atomic Force Microscopy (AFM)

**Figure 1:**
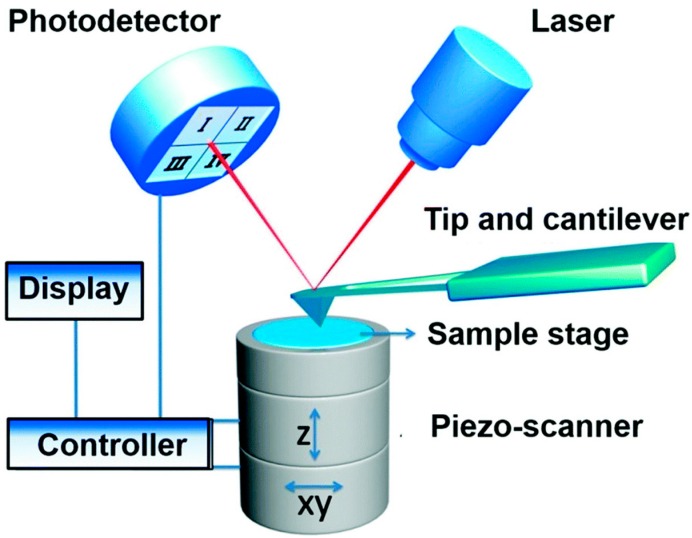
Schematic diagram of atomic force microscopy. In AFM, the tip-sample interactions are detected to characterize the topography and biophysical properties of sample. Reproduced from [2] with permission.

AFM is a topographic imaging technique with high spatial resolution (a lateral resolution of 1 nm and a vertical resolution of 0.1 nm), and can be used to acquire mechanical properties [16]. The principle of AFM (Figure 1) is that the sample can be imaged at atomic resolution by detecting the near-field interactions between a tiny tip and the sample surface [17,18]. There are two fundamental modes (contact mode and tapping mode) for AFM imaging. For contact mode imaging, the tip on a cantilever is brought into gentle contact with the sample and then raster scanned over the sample surface; by maintaining a constant force on the tip, the tip-sample interactions will induce the deformation of the cantilever which can be detected by a photodetector and converted to an electrical signal read by a computer and, thus, the sample topography is recorded [2,18]. Tapping mode imaging is similar to contact mode imaging except that the piezo (acoustic) or magnetic coil (magnetic) provides a constant driving force causing the tip to oscillate at a certain frequency [19]. Compared to contact mode, tapping mode can reduce the lateral forces on the tip and thereby minimize deformation of soft samples.

In addition to imaging, AFM-based force spectroscopy is also a versatile approach to measure the interaction forces of biological systems [20]. In this mode, the AFM force curve is obtained by recording the cantilever deflection while the tip approaches the surface and withdraws from it [21]. The force curve can be used to extract information, such as stiffness, elasticity, and molecular interactions [22,23]. Single-molecule force spectroscopy (SMFS) is used to measure the forces of the interaction between individual ligands and receptors. To measure the molecular recognition forces, the tip is functionalized with specific ligands via a bifunctional crosslinker that can distinguish specific ligand-receptor interactions from unspecific interactions [20]. SMFS has made plenty of progress in the research of the interactions between individual biomolecules, such as molecular recognition between antibodies and antigens, drugs and receptors, and complementary strands of DNA [4,24]. Recently, force-distance curve-based AFM has been developed to achieve multiparametric imaging at nanometer resolution. Through recording an array of force-distance curves and real-time extracting the parameters of physical properties, force-distance curve-based AFM can be used to acquire the sample topography and, meanwhile, map the distributions of multiple physical properties [25].

### 2.2. Conventional Fluorescence Microscopy

#### 2.2.1. Confocal Laser Scanning Microscopy (CLSM)

**Figure 2:**
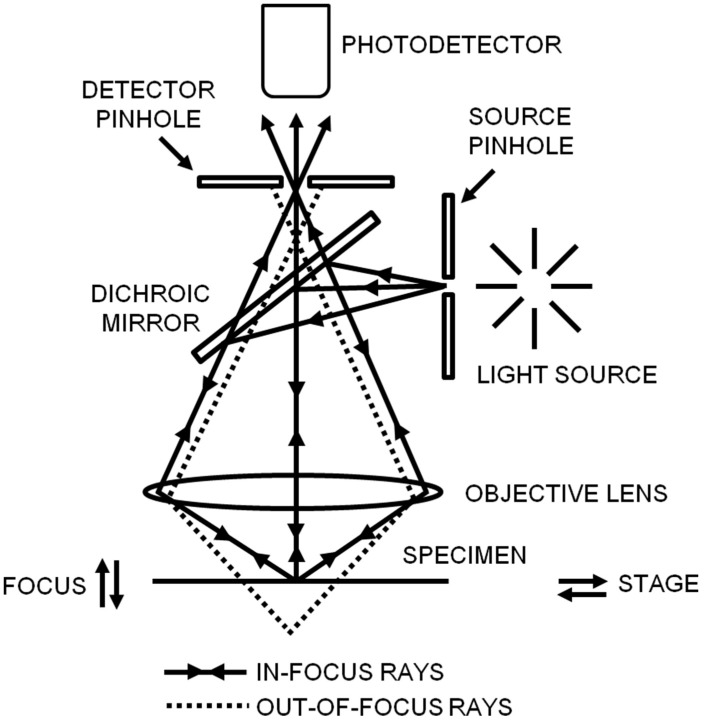
Schematic diagram of confocal laser scanning microscopy. In confocal microscopy, the point illumination, the detector pinhole, and the focus in the specimen are all confocal with each other. Reproduced from [28] with permission.

Since confocal microscopy has a non-invasive ability and can penetrate deep into a sample, it has been applied to many scientific fields, such as drug delivery and virus invasion [26,27]. The principle of confocal microscopy is shown in Figure 2; in confocal microscopy, the illumination and detection are restricted to a diffraction-limited area in the sample, which rejects a higher background and yields a higher resolution in contrast to wide-field epifluorescence microscopy [28,29]. To achieve this goal, the point illumination is confocal with the pinhole in front of the photodetector; when the sample is simultaneously positioned in the plane of focus of the point illumination and the pinhole, the emitted light from the focal plane can pass through the pinhole, but light from above or below the focal plane will be hindered by the pinhole. Although confocal microscopy enhances the imaging resolution, the field of view is limited, and thereby it needs to be combined with a scanning technique to acquire a whole image and achieve three-dimensional imaging. To improve the speed of acquiring confocal images, disk-scanning confocal microscopy is developed which uses a disk (Nipkow disk or spinning-disk) including multiple pinholes to scan the sample [30]. Moreover, multi- or two-photon excitation microscopy is developed for imaging thick samples (1 mm) along with the reduced phototoxicity. In multi- or two-photon microscopy, the pinhole in front of the photodetector is not necessary since the excitation and the emitted light only occurs in focus [31].

#### 2.2.2. Total Internal Reflection Fluorescence Microscopy (TIRFM)

**Figure 3:**
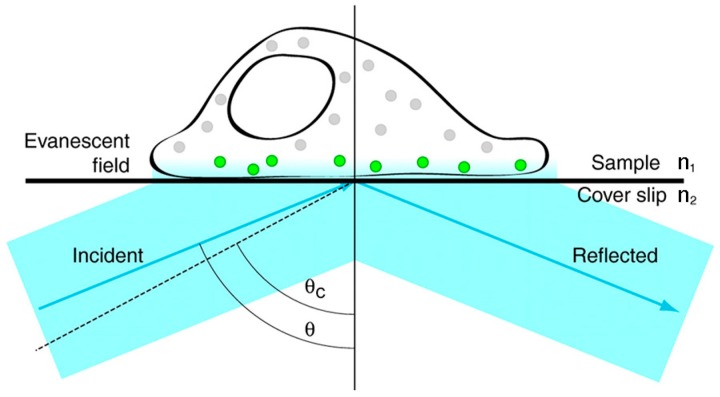
Schematic diagram of total internal reflection fluorescence illumination. When the excitation beam travels across the coverslip-sample interface (n_1_ < n_2_) with an incident angle θ above the critical angle θ_c_ (indicated by the dashed line), the excitation beam is totally internally reflected back into the cover slip and an evanescent field is generated in the sample. Only fluorophores that are located in the evanescent field are excited (indicated by the green color). Here, n_1_ and n_2_ are, respectively, the refractive indices of the sample and the glass coverslip. Reproduced and rearranged from [34] with permission.

TIRFM is quite suitable for investigating dynamic biological processes, such as endocytosis and cytoskeleton remodeling occurring near the cell membrane [32,33]. Figure 3 shows the basic principle of TIRFM. When the excitation light propagates from a medium of high refractive index (RI) (e.g., cover slip) to a low RI medium (e.g., a cell) at a high incident angle θ (greater than the critical angle θ_c_), it is totally internally reflected back into the cover slip and generates a thin evanescent field (within 100 nm) in the low RI medium. The evanescent field has the ability to selectively excite fluorophores near the interface and reduce background fluorescence due to the intensity of the evanescent field exponentially decaying away from the interface [34,35]. Therefore, TIRFM is a useful tool to provide a high-contrast image of biomolecules stained with fluorochromes near the cell surface. Compared with confocal microscopy, TIRFM has a higher temporal resolution and a higher signal-to-noise ratio, but TIRFM is confined to imaging the biological molecules at or near the cell membrane [35].

### 2.3. Super-Resolution Fluorescence Microscopy

#### 2.3.1. Stimulated Emission Depletion (STED) Microscopy

**Figure 4:**
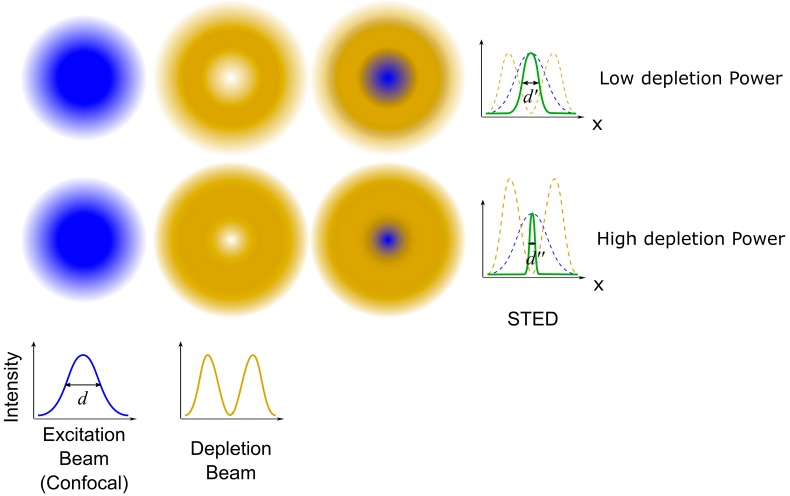
The basic principle of stimulated emission depletion microscopy. In STED, the depletion beam is superimposed to the excitation beam to reduce the size of the excitation spot. The higher the depletion beam power, the smaller the size of the excitation spot. Reproduced and rearranged from [36] with permission.

STED is a super-resolution imaging technique based on the platform of confocal microscopy, and can be used to image biological samples with resolutions of about 40 nm in both lateral and axial directions [36]. The basic principle of STED is to inhibit fluorescence at the rim of the excitation spot by superimposing the excitation beam with a donut-shaped depletion beam (Figure 4). When the depletion beam has a wavelength included in the emission spectrum of the dye and the intensity beyond the saturation intensity, it can stimulate all of the excited electrons to relax at the same wavelength of the depletion beam, thereby allowing the fluorescence in the areas where the depletion beam falls to be switched off by a spectral filter that rejects photons at the wavelength of the depletion beam. In other words, the size of the excitation spot can be controlled below the diffraction limit by the depletion beam, and it can be further reduced while increasing the depletion beam intensity. Hence, the super-resolution image can be generated by the two overlaid beams (the excitation beam and the depletion beam) raster scanning across the sample point by point [37,38,39].

#### 2.3.2. Single-Molecule Localization Microscopy (SMLM)

**Figure 5:**
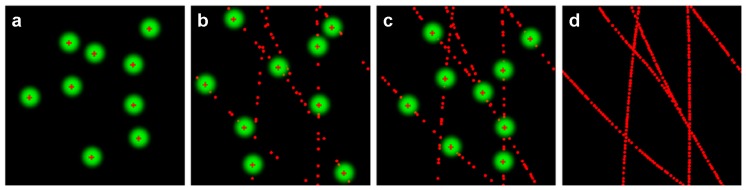
Imaging principle of single-molecule localization microscopy. In SMLM, only a small subset of fluorophores can be randomly switched on using appropriate illumination and localized at high resolution; after the small subset of fluorophores is switched off, a new subset is switched on and localized. This cycles is repeated to record many frames, including the localizations of individual fluorophores (**a**–**c**). Therefore, an super-resolution image is reconstructed from all of the successful localizations (**d**). Reproduced and rearranged from [36] with permission.

Recently, single-molecule localization microscopy (e.g., stochastic optical reconstruction microscopy (STORM) and photoactivated localization microscopy (PALM)) has been developed [10,11,40] and provides an unprecedented opportunity to investigate cell membrane ultrastructures at the single-molecule level [41,42]. Figure 5 shows the imaging principle of SMLM; SMLM is based on the positions, with single-molecule precision, of each fluorophore in the sample to reconstruct a super-resolution image [36,43,44]. For SMLM imaging, photoswitchable fluorescent probes (e.g., photoswitchable dyes for STORM and photoactivatable fluorescent proteins for PALM), which have the property of switching between non-fluorescent and fluorescent states by illumination with an appropriate light, are required to label samples [45,46]. During the process of image acquisition, all of the probes are firstly quenched; then, using appropriate illumination, a sparse subset of probes can be randomly activated and imaged; once these probes are deactivated, a new subset is activated and imaged. By repeating the cycles of activation and deactivation many frames, with each frame containing the positions of several individual probes, are recorded. After image acquisition, the localizations of these single probes are determined with very high resolution by finding the centroid of the point spread function; by superimposing all of the successful localizations, a super-resolution image of the sample is constructed.

## 3. Correlative Optical Microscopy/Atomic Force Microscopy

### 3.1. Correlative Conventional Fluorescence Microscopy/Atomic Force Microscopy

Correlative AFM and confocal microscopy has been increasingly used to investigate biological structures and processes. By correlating the AFM topographic image with the corresponding fluorescence image, Burns investigated the lateral structure of lipid domains in model membrane bilayers [47]. Frankel et al. also used correlative AFM and confocal microscopy to investigate protein domains at the cytoplasmic side of the plasma membrane. The authors observed that the irregularly-shaped protein domains seen in AFM topography included both resting and activated immunoglobin E receptors (FcεRI), aggregated GM1 ganglioside, and clathrin, and they found that the protein domains were related to cholesterol and the cytoskeleton. Taken together, these results suggested that both cholesterol and the cytoskeleton contribute to the formation of the protein domains that were vital for cell functions, such as signaling and endocytosis [48]. To reveal the structure of focal adhesion complexes in quasi-native states, Franz and Muller combined AFM with confocal microscopy to image focal adhesions which were prepared by de-roofing fibroblasts with short ultrasonic bursts. They observed that microfilament bundles in focal adhesions were predominantly arranged in a parallel pattern and occasionally branched. By correlating the AFM height information with fluorescence intensities, they found that paxillin and F-actin respectively localized in the membrane-proximal and membrane-distal half of focal adhesions [49].

**Figure 6:**
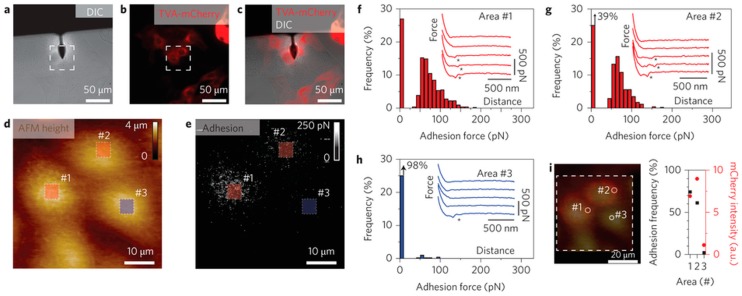
Imaging a virus binding to cells using correlative force-distance curve-based AFM and confocal microscopy. The AFM tip was functionalized with a single EnvA-RABV (∆*G: eGFP*) virus (EnvA: the glycoprotein of the avian sarcoma leukosis vieus subgroup A; RABV: rabies virus). Mixed cultures of wild-type MDCK cells and TVA-mCherry (TVA: the avian tumor virus receptor A) expressing MDCK cells (red) were grown for three days. DIC image (**a**), fluorescence image (**b**) and overlay of both images (**c**) guiding the AFM tip to choose an area of interest (the dashed square), including both cell types. AFM topography (**d**) and corresponding adhesion map (**e**) in the dashed square, which were used to evaluate specific and nonspecific virus binding events. Distribution of adhesion forces of specific interactions (**f**,**g**) and nonspecific interactions (**h**). (**i**) Merged image of the topography and fluorescence images. The adhesion frequency was in line with the relative fluorescence intensity, which meant the specific adhesion events corresponding to specific binding events. Reproduced from [53] with permission.

In terms of the advantages of correlative CLSM/AFM, it cannot only correlate the topographic information with the information from fluorescence image, but can also couple the mechanical properties provided by AFM with the specific components identified by the confocal microscopy. Haga et al. used the correlative CLSM/AFM technique to correlate the AFM topography, elasticity map reconstructed from force curves, and the fluorescence images of actin filaments, intermediate filaments, and microtubules in a living fibroblast, which revealed that the cellular elasticity was closely related to the distributions of both the actin network and intermediate filaments [50]. In correlative CLSM/AFM microscopy, the AFM tip can be used as a micromanipulator to stimulate cells, and the confocal microscopy is used as a detector to reveal the cellular responses [51]. Recently, Hecht et al. developed a uniaxial cell stretcher to integrate into a correlative CLSM/AFM system. Therefore, this unique tool can be used to simultaneously detect cellular responses with real-time AFM and fluorescence studies while stretching cells [52]. Based on correlative force-distance curve-based AFM and confocal microscopy, Alsteens and coworkers simultaneously quantified the first viral binding events while imaging living cells. By fluorescently labelling the virus and the corresponding receptors expressed in cells, and functionalizing the AFM tip with the labelled virus, the authors could precisely localize and quantify the individual virus binding events (Figure 6). They also contoured the free-energy landscape for the first virus-receptor binding events [53]. The combination of colloidal probe AFM and confocal microscopy can be used to investigate the relationships between mechanical properties and the release of microcapsules that were useful for drug delivery [54,55].

**Figure 7:**
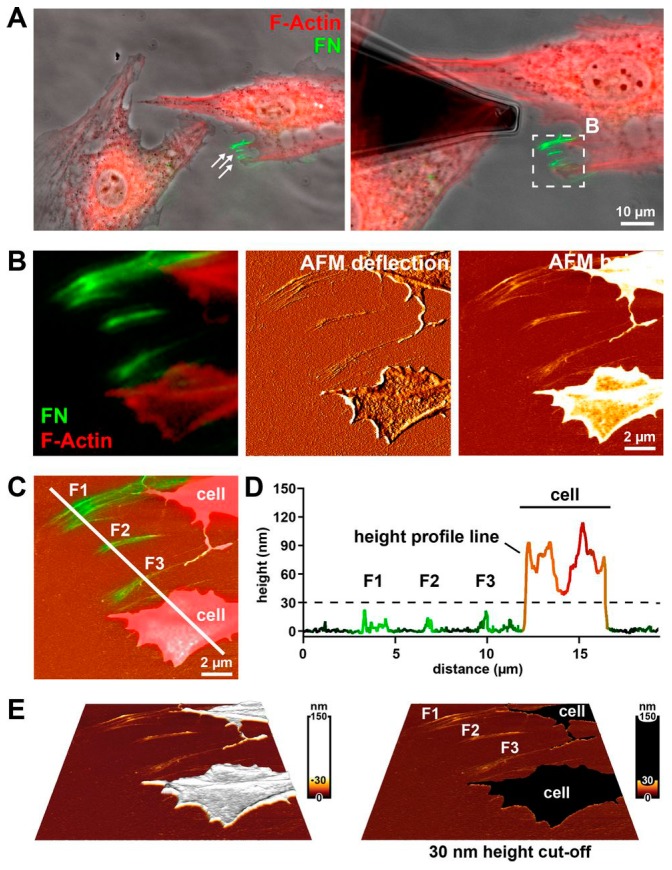
Imaging mature fibronectin (FN) fibril structure using the correlative TIRFM/AFM technique. In this experiment, the F-actin (red) of rat embryonic fibroblasts (REF52) were stained to visualize cellular structure, the live REF52 cells were incubated on a homogeneous coating of Alexa 488-labelled FN (green) for 4 h, and then fixed before data acquisition. (**A**) Superimposition of the phase contrast image and the fluorescence image guiding the AFM tip to choose a region including FN fibrils. (**B**) Three correlative images (fluorescence image, AFM deflection image, and AFM topography) of the same region in the dashed square of (**A**). (**C**) The merged image of topography and fluorescence image showing the FN fibril structure and the cellular structure. (**D**) Correlating the height with the corresponding fluorescence intensity to distinguish the FN fibril structure and the cellular structure using a 30-nm height cut-off (dashed line). (**E**) Three-dimensional topography intuitively showing the FN fibril structure. Reproduced from [57] with permission.

TIRFM has the advantage to image biological phenomena happening at, or near, the interface with a higher axial resolution compared to confocal microscopy. Thereby, correlative TIRFM/AFM microscopy is well suited for correlating the topographic information with the distribution of specific components at the interface. Brown et al. applied correlative TIRFM/AFM to investigate self-assembled myosin filaments under liquid environments. By quantitatively correlating the height and the fluorescence intensity of the self-assembled myosin filaments, they confirmed that the myosin heads are arranged in a shell of roughly constant thickness around the filament [56]. Recently, Gudzenko and Franz combined time-lapse AFM and TIRFM to study the dynamics of fibronectin fibrillogenesis in living cells at early stages. The authors respectively recorded correlative topography/fluorescence images of fixed cells which were incubated on fluorescently-labelled fibronectin for 4 and 16 h (Figure 7), and thereby acquired the structure information of fibronectin. Based on the fibronectin structure, they monitored the early fibrillogenesis dynamics at high resolution by time-lapse AFM, and they also found that Mn^2+^ enhanced the early fibrillogenesis [57]. Oreopoulos et al. reported the coupling of AFM and polarized TIRFM technique, which can correlate the topographic features and the information (e.g., molecular orientation, conformation and rotational mobility) provided by polarized TIRFM [58,59].

Correlative TIRFM/AFM microscopy also has the ability to monitor real-time sub-cellular changes in response to mechanical force, which is vital for understanding mechanically-induced cellular remodeling. Using correlative TIRFM/AFM, Mathur et al. investigated stress transmission in endothelial cells. When stimulating the apical membrane with a force of 0.3–0.5 nN, they observed global focal contact rearrangements on the basal surface, which suggested that cells transfer localized force from the apical membrane to the basal membrane globally [60]. Trache and Lim integrated AFM with TIRFM and fast-spinning disk (FSD) confocal microscopy to study live cell response to mechanical stimulation in real-time [61]. Using this combined AFM/TIRFM/FSD confocal microscopy, Lim and coworkers investigated live vascular smooth muscle cells’ (VSMC) response to tensile stress using the AFM tip functionalized with extracellular matrix (ECM) proteins. By integrating mechanical stimulation with simultaneous TIRFM imaging and spinning-disk confocal imaging, they found that VSMC could adaptively respond to mechanical stimulation by the actomyosin apparatus modulation [62].

### 3.2. Correlative Super-Resolution Fluorescence Microscopy/Atomic Force Microscopy

**Figure 8:**
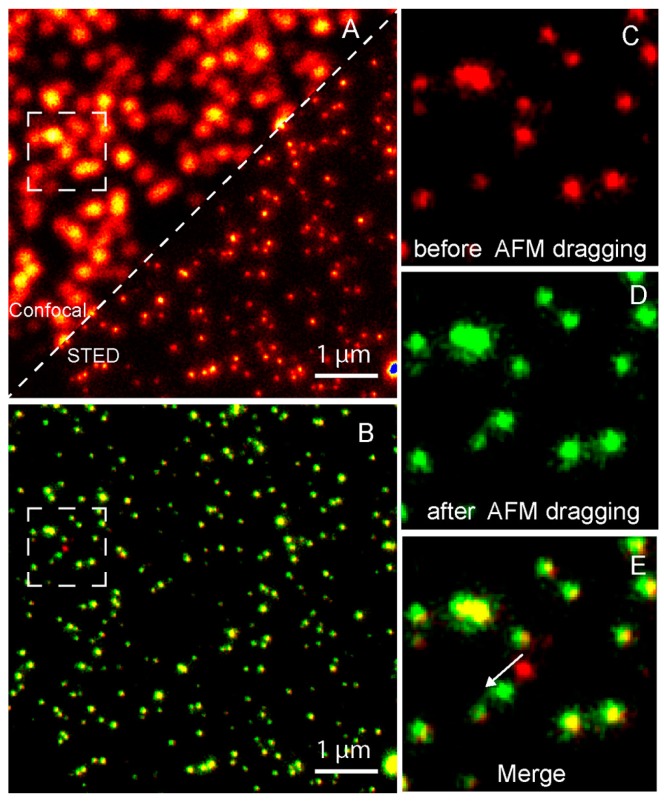
Nanomanipulation of a 40 nm fluorescent bead using the correlative STED/AFM technique. (**A**) A comparison of confocal and STED images which shows that STED has a higher resolution. (**B**) Merged image of STED images acquired before (red) and after (green) AFM dragging of the same area. The overlay of both colors shows stationary beads in yellow. Magnified STED images acquired before (**C**) and after (**D**) AFM dragging, and the corresponding merged image (**E**), which clearly shows that the movement made by AFM at a subdiffraction distance. Reproduced from [64] with permission.

Although correlative conventional fluorescence microscopy/AFM techniques have gained great achievements in the studies of biological questions, ranging from lipid bilayer structure and membrane protein assembly to cellular structure, function, and dynamics, the resolutions of both techniques are not well aligned, and thereby these correlative techniques are not well suited to investigate molecular localization and dynamics in cells at the single-molecule level. To correlate topography with fluorescence specificity at the single-molecule level, it is inevitable to combine AFM with super-resolution fluorescence microscopy. Diaspro and coworkers developed a novel nanoscopic tool by coupling AFM with STED for the first time, which is capable of correlating the topographic image, super-resolution fluorescence image, and a force map, such as Young’s modulus. They confirmed the device performance using fluorescent spheres of 40 nm size and fluorescently-labelled microtubules in cos7 cells [63]. The correlative STED/AFM technique also has the ability to simultaneously nanomanipulate specific molecules of interest and monitor the manipulation effects with a resolution of tens of nanometers. Figure 8 shows an example of nanomanipulation using the correlative STED/AFM technique. The AFM tip dragged only one specific 40 nm fluorescent sphere on the Poly-l-lysine-coated coverslip with the help of STED monitoring [64]. Diaspro et al. also achieved bionanomanipulation using the correlative STED/AFM technique, which might be useful for nanosurgeries [65].

**Figure 9:**
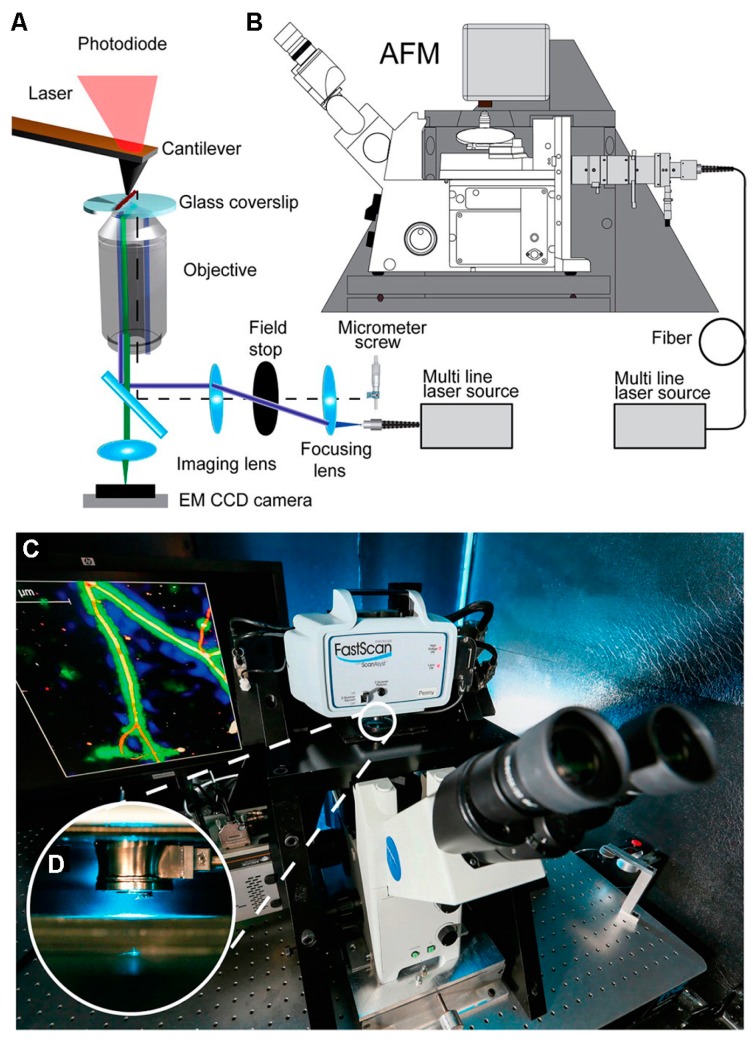
The setup of correlative SMLM/AFM microscopy. (**A**) Schematic of the optical path which is aligned with the AFM cantilever. (**B**) Schematic of the AFM integrated with an inverted optical microscope. (**C**) Photograph of the correlative SMLM/AFM instrument. (**D**) Magnified photograph showing the AFM cantilever aligned with the optical axis. Reproduced from [15] with permission.

Compared to STED, single-molecule localization microscopy is capable of precisely identifying single molecules and quantitative imaging, but has a limited temporal resolution. Therefore, the combination of AFM and single-molecule localization microscopy is highly suitable for correlating nanoscale topography with precise localization of a specific molecule. Diaspro et al. respectively used STED/AFM and STORM/AFM to image α-tubulin in Hela cells; they found that both of the correlative techniques could correlate morphological features, super-resolution fluorescence imaging, and mechanical properties, and had their own advantages [66]. Correlative AFM and single-molecule localization microscopy also has the advantage to eliminate artefacts that result from poor labelling and image reconstruction [67]. Recently, Odermatt et al. built a correlative SMLM/AFM microscopy by integrating an AFM and an inverted optical microscope (Figure 9). In this device, the optical path was aligned with the AFM cantilever. They first confirmed the performance of the correlative microscopy via imaging polymerized actin filaments on a coverslip with both imaging modalities (AFM imaging and STORM imaging); then, they validated that the correlative microscopy can be used to image live mammalian cells, thereby providing a new method for the investigations of structure-function relationships at the single-molecule level [15].

## 4. Challenges and Outlook

Combining AFM with optical microscopy has been successfully used to correlate the structure with the physiological function of specific components under close to native conditions. However, correlative AFM and optical microscopy still have many challenges to be solved. To achieve truly meaningful correlation between AFM and optical microscopy, it is necessary to consider resolution matching of the two techniques. Although correlative AFM and conventional fluorescence microscopy has achieved great success in overcoming the shortcomings of both techniques, the unmatched resolution between AFM and conventional fluorescence microscopy produces difficulty in correlating the structure information with the distribution of specific components at the single-molecule level. To fully utilize the advantages of fluorescence microscopy, super-resolution fluorescence microscopy achieving nanometer resolution is combined with AFM.

For correlative AFM and super-resolution fluorescence microscopy, the challenges include sample preparation, imaging speed and image alignment. To maintain optimal performance of both techniques, it is necessary to optimize sample preparation protocols. First of all, we choose a glass coverslip which meets the imaging requirements (good optical properties for super-resolution fluorescence microscopy imaging, and flatness for AFM imaging) of both techniques as the substrate. Then we need to consider the fluorescent labelling strategy; to avoid altering the structure of samples, using small molecules, such as fluorescently-labeled aptamer/peptide [68,69], or fusion proteins [11] is necessary. Additionally, the excitation spectra of fluorophores must be away from the AFM laser to avoid bleaching these fluorophores. Particularly, the imaging buffer for STORM degrades samples [15], which can limit the combination of STORM and AFM to achieve a truly synchronized correlation. Therefore, it is necessary to further develop small molecule markers and optimize the imaging buffer for both AFM and super-resolution fluorescence microscopy.

For imaging speed, AFM can achieve the temporal resolution of minutes which is comparable to that of SMLM. Due to the limit of temporal resolution, correlative AFM/SMLM is not suited for investigating cellular dynamics at nanometer resolution. Recently, super-resolution fluorescence microscopy has been developed to achieve second and subsecond temporal resolution [70,71], which is comparable to that of high-speed AFM [72]. Therefore, combining a high-speed AFM with a super-resolution fluorescence microscopy that has the advantage of quickly acquiring image will pave a new way to study cellular dynamics at the single-molecule level. To correlate the results from both AFM and super-resolution fluorescence microscopy, it is indispensable to overlay images obtained by correlative microscopy with a precision down to 10 nm. To achieve this goal, we need to optimize procedures to align these correlative images according to the identical structure of these correlative images or fiduciary markers, such as quantum dots [15]. In a word, we need to substantially improve and enhance both AFM and super-resolution fluorescence microscopy and then integrate the two techniques to achieve a high degree of matching and synchronized co-localization measurement, which is adapted to the developing requirements of life sciences.

## 5. Conclusions

Nowadays, AFM has gradually evolved into a multifunctional tool [17]. AFM can not only provide three-dimensional topography with lateral and axial resolutions of 1 nm and 0.1 nm, respectively, but also determine the mechanical properties with high sensitivity of tens of piconewton forces. Furthermore, AFM can be used to manipulate biological samples at subnanometre resolution. However, it is difficult to simultaneously identify two or more specific components using the AFM technique. Therefore, combining AFM with other techniques, particularly optical fluorescence microscopy, which can give excellent specificity, is becoming inevitable to complement the shortcomings of the individual technique.

The combination of AFM and conventional fluorescence microscopy has been reported, such as correlative CLSM/AFM and TIRFM/AFM. Although conventional fluorescence microscopy techniques have the advantage of specificity, they are limited by the imaging resolution: 250 nm in the lateral direction and 500 nm in the axial direction [8]. Due to the resolution of conventional fluorescence microscopy is incompatible with that of AFM, the combination of both techniques cannot achieve the truly synchronized co-localization measurement. Recently, super-resolution fluorescence microscopy techniques have been developed and have broken the diffraction barrier, achieving nanometer resolution [12]. Hence, combining AFM with super-resolution imaging techniques can complement the strengths and weaknesses of both techniques. Several correlative AFM and super-resolution fluorescence microscopy techniques, such as correlative STED/AFM, PALM/AFM, and STORM/AFM, have been developed to correlate morphological information with specificity at nanometer resolution. However, the combination of AFM and super-resolution fluorescence microscopy is still in the early stage and has many problems to be solved, such as sample preparation, imaging speed, and image processing. Although there are still many problems for these correlative techniques, the correlative techniques open new avenues for the investigations of biological systems at the single-molecule level.
